# Supplementary material for: CD276 as a Candidate Target for Immunotherapy in Medullary Thyroid Cancer
Source: Int J Mol Sci. 2023 Jun 12;24(12):10019. doi: 10.3390/ijms241210019 (PMC10298428; doi:10.3390/ijms241210019)
Supplement: Supplementary file 1 [file ijms-24-10019-s001.zip › STROBE checklist.pdf]

STROBE checklist

**Title and abstract**	CD276 as a candidate target for immunotherapy in medullary thyroid cancer
	Medullary thyroid cancer (MTC) is a rare malignancy, and the treatment of metastatic MTC is challenging. In previous work, immune profiling of MTC identified CD276 as a potential target for immunotherapy. CD276 expression was 3-fold higher in MTC cells than in normal tissues. Paraffin blocks from patients with MTC were analyzed by immunohistochemistry to confirm the results of RNA-Seq. Serial sections were incubated with anti-CD276 antibody, and scored ac-cording to staining intensity and the percentage of immunoreactive cells. The results showed that CD276 expression was higher in MTC tissues than in controls. A lower percentage of immunoreactive cells correlated with the absence of lateral node metastasis, lower levels of calcitonin after surgery, no additional treatments, and remission. The intensity of immunostaining and the percentage of CD276 immunoreactive cells were statistically significantly associated with clinical factors and the course of the disease. These results suggest that targeting this immune checkpoint molecule CD276 could be a promising strategy for the treatment of MTC.
Background/rationale	Medullary thyroid cancer (MTC) is the third most common thyroid cancer, ac-counting for 3–5% of all thyroid cancer cases. Surgery plays a major role in the treatment of MTC. Metastasis to central and latero-cervical lymph nodes occurs in up to 90% of patients with tumors >4 cm in diameter. Distant metastases are present at diagnosis in approximately 10% of MTC patients and detected at a higher rate (19–38%) during follow-up. The treatment of patients with locally advanced or metastatic disease remains problematic. The 10-year overall survival (OS) rate in unselected MTC patients is approximately 75%, whereas it is 40% in patients with locally advanced or metastatic disease. Patients with locally advanced disease are treated with external beam radiation therapy (EBRT), whereas those with metastatic disease are treated with tyrosine kinase inhibitors (TKIs), which increase progression-free survival but do not improve OS. However, resistance to approved drugs has been reported. Although the use of immunotherapy for MTC has been investigated, there are no approved drugs to date.
Objectives	Evaluation the correlation between expression of CD276 and immunohistochemistry (IHC) staining intensity with histo-clinical features in MTC.
Study design	Analysis of paraffin blocks from patients with MTC by immunohistochemistry to confirm the results of RNA-Seq. Serial sections were incubated with anti-CD276 antibody, and scored ac-cording to staining intensity and the percentage of immunoreactive cells.
Setting	The study was conducted in Holycross Cancer Centre, Kielce (HCC). Archival paraffin blocks of MTC were obtained from then Pathology Department of Holycross Cancer Center. All study procedures were approved by the Institutional Review Board of Jan Kochanowski University, Kielce, Poland (approval number: 12/2020).
Participants	*Cohort study*— The study included 46 patients with MTC who were selected from the thyroid cancer database at the Endocrinology Clinic of Holycross Cancer Centre, Kielce (HCC). The initial response to therapy was evaluated at 3 months after surgery. Responses were classified as excellent, biochemical incomplete, or structural incomplete. The final follow-up was on December 31st, 2021, and the patients were placed in the following categories: no evidence of disease (NED), biochemical incomplete, structural incomplete, death/MTC-related, and death/MTC-unrelated.<br>*Case-control study*— Benign thyroid tumors
Data sources/ measurement	Serial sections of formalin fixed, paraffin-embedded tissue samples were stained with monoclonal anti-CD276 antibody (Invitrogen, 6A1). Briefly, staining was performed on the Ventana BenchMark XT (Ventana Medical Systems Inc.). The staining protocol included

	online deparaffinization, HIER (Heat Induced Epitope Retrieval) with Ventana Cell Conditioning 1 for 32 min, and primary antibody incubation for 20 min at 31°C. Antigen-antibody reactions were visualized using the Ventana OptiViewTM Amplification kit, followed by Ventana OptiViewTM Universal DAB Detection Kit (Optiview HQ Linker, 8 min; Optiview HRP Multimer, 8 min; Optiview Amplifier H2O2/Amplifier, 4 min; Optiview Amplifier Multimer, 4 min; Optiview H2O2/DAB, 8 min; Optiview Copper, 4 min). Counterstaining was performed using Ventana Hematoxylin II for 8 min, followed by bluing reagent for 4 min. Finally, slides are removed from the stainer, dehydrated, and coverslipped for microscopic examination. Positive controls included a known CD276 positive human tonsil tissue. The scoring criteria for staining were based on the intensity of immunostaining and the percentage of immunoreactive cells, and the percentage of CD276-positive cells and the intensity of the staining were scored. IHC-CD276 staining expression was scored as "weak" (1+), "moderate" (2+), and "strong" (3+), and samples were divided into three groups according to the percentage of CD276-positive cells as follows: 11–50%, 51–75%, and 76–100%; <10% positive cells was considered a negative result.<br><br>Thirteen samples (28.3%) had a staining intensity of 1+. Intensity of immunostaining 2+ and 3+ was observed in 24 (52.2%) and 9 (19.6%) samples, respectively. The number of patients and the percentage of CD276-positive cells were as follows: 20 (43.5%) for 11–50%, 21 (45.7%) for 51–75%, and 5 (10.9%) for 76–100%. No CD276 immunoreactive cells were observed in the tissues outside the tumor.
Study size	All those with a diagnosis of MTC treated at the Endocrinology Clinic of Holycross Cancer Center, Kielce (HCC) were included in the study.
Statistical methods	Continuous data were expressed as medians, quartiles, and range (minimum and maximum). Categorical data were summarized by frequencies and percentages. Group comparisons were performed using the chi-square test or Fisher's exact test for categorical variables and the Mann U-test for quantitative variables (because of the lack of normality in the Shapiro-Wilk test).<br>Statistical tests were two-tailed and a p-value <0.05 was considered significant. All statistical analyses were performed using R (version 4.0.3; The R Foundation for Statistical Computing, Vienna, Austria).
Participants	The study included 46 patients with MTC (42 living and four dead patients).
Descriptive data	The study group comprised 31 women (67.4%, 31/46) and 15 men (32.6%, 15/46). The median age at diagnosis was 52 years (range, 24–84). The median tumor size was 14 mm (range, 2–100). Most patients (69.6%, 32/46) did not have tumor multifocality. Gross type extrathyroidal extension was detected in 6.5% of patients (3/46). Angioinvasion was present in 10.9% (5/46) of patients. Lymph node metastases and distant metastases were present in 51.3% (19/46) and 4.3% (2/46) of patients, respectively.
Key results	Univariate analysis suggested statistically significant correlations of higher intensity of immunostaining (2+ or 3+) and the percentage of immunoreactive cells for CD276 (51–100%) with gender, lateral lymph node metastasis, and postoperative serum CT levels.<br>Increased expression of CD276 was observed more frequently in men ($p = 0.0255$). Lateral lymph node metastasis ($p = 0.0010$) was more frequent in patients with 2+ or 3+ intensity of immunostaining and 51–100% of CD276 immunoreactive cells. A 3-fold higher number of patients with increased CD276 expression postoperatively had serum CT levels ≥2 ($p = 0.0036$). More than 2 times more germline mutations in the RET gene occurred among patients with 2+ or 3+ intensity of immunostaining and 51-100% of CD276 immunoreactive cells ($p = 0.02321$). A less than excellent response to initial therapy was

	observed in 22 patients, and this was significantly associated with 2+ or 3+ intensity of immunostaining and 51–100% of CD276 immunoreactive cells (p = 0.0374). Furthermore, statistical analysis showed that patients from the 51–100% CD276-positive group less frequently achieved disease remission (p = 0.0053) and more frequently required additional therapies (p = 0.0293). No statistically significan correlation was observed with any other features analyzed.
Limitations	The size of the study group and the single-center nature of the study, the results obtained may not be representative of the population.
Interpretation	In this study group, CD276 was expressed in all MTC samples. Because of the low number of C cells in the healthy thyroid gland and their diffuse occurrence, CD276 was not detected in normal cells. CD276 was initially characterized as a T cell stimulating protein, although most current studies describe it as a T cell inhibitor that promotes tumor aggressiveness and proliferation. Thus, CD276 may be an important immunological target in cancer. The role of CD276 in the development of MTC and during its clinical course has not been evaluated to date. In this study, we showed that a higher immunostaining intensity and a greater percentage of CD276 immunoreactive cells were associated with certain clinical factors related to a severe course of the disease, response to therapy, and the status at the end of treatment. Assessment of the relationship between CD276 expression in tumor cells and clinical factors demonstrated its effect on lateral lymph node metastasis and postoperative serum CT levels, underscoring the need to identify effective therapies for patients with MTC and elevated CD276 protein levels.We observed significant correlations between CD276 staining intensity and clinicopathological features. The involvement of the CD276 pathway in immune responses and MTC development needs to be further investigated to design effective treatments for patients with MTC.
Generalisability	CD276 influences the development and course of MTC. In addition, it is worth noting that CD276 plays a role in the development of many types of cancer, including breast cancer, lung cancer, ovarian cancer, brain tumors, gastric cancer, and squamous cell carcinoma) and its elevated expression correlates with a worse clinical course. Therefore, CD276 can be considered as a versatile candidate for immunotherapy, capable of being used as a molecular target in the treatment of many types of cancer.
Funding	This research was funded by the Minister of Education and Science called “Regional Initiative of Excellence” in the years 2019-2022, project no. 024/RID/2018/19, amount of financing 11 999 000,00 PLN.
