# Supplementary material for: CD276 as a Candidate Target for Immunotherapy in Medullary Thyroid Cancer
Source: Int J Mol Sci. 2023 Jun 12;24(12):10019. doi: 10.3390/ijms241210019 (PMC10298428; doi:10.3390/ijms241210019)
Supplement: Supplementary file 1 [file ijms-24-10019-s001.zip › Supplementary Table S1.pdf]

Supplementary Table S1. Clinical characteristics of patients according to staining intensity and percentage of immunoreactive cells with CD276 expression.

	IHC-CD276 (1+ or 2+) (11–50% cells)	IHC-CD276 (2+ or 3+) (51–100% cells)	Total	*p*-value
	n=20	*n*=26	*n*=46	
Gender, *n* (%)				0.0255
Female	17 (85.0%)	14 (53.8%)	31 (67.4%)	
Male	3 (15.0%)	12 (46.2%)	15 (32.6%)	
Median age at diagnosis, years (Q1,Q3)	50.5 (40.5, 65.5)	54.5 (44.5, 60.8)	52.0 (41.0, 61.8)	0.9117
Median tumor size, mm (Q1,Q3)	12.5 (6.0, 20.0)	14.0 (8.2, 24.5)	14.0 (7.0, 23.0)	0.5789
Multifocality, *n* (%)				0.9552
No	14 (70.0%)	18 (69.2%)	32 (69.6%)	
Yes	6 (30.0%)	8 (30.8%)	14 (30.4%)	
Extrathyroidal extension, *n* (%)				0.5718
No	18 (90.0%)	25 (96.2%)	43 (93.5%)	
Yes	2 (10.0%)	1 (3.8%)	3 (6.5%)	
Angioinvasion, *n* (%)				1
No	18 (90.0%)	23 (88.5%)	41 (89.1%)	
Yes	2 (10.0%)	3 (11.5%)	5 (10.9%)	
Tumor stage, *n* (%)				0.7878
T1a	6 (30.0%)	6 (23.1%)	12 (26.1%)	
T1am	4 (20.0%)	4 (15.4%)	8 (17.4%)	
T1b	4 (20.0%)	5 (19.2%)	9 (19.6%)	
T1bm	1 (5.0%)	2 (7.7%)	3 (6.5%)	
T2	3 (15.0%)	3 (11.5%)	6 (13.0%)	
T2m	1 (5.0%)	3 (11.5%)	4 (8.7%)	
T3	0 (0.0%)	3 (11.5%)	3 (6.5%)	
T3m	1 (5.0%)	0 (0.0%)	1 (2.2%)	

Node stage, *n* (%)				0.0021
N0	15 (75.0%)	12 (46.2%)	27 (58.7%)	
N1a	4 (20.0%)	1 (3.8%)	5 (10.9%)	
N1b	1 (5.0%)	13 (50.0%)	14 (30.4%)	
Lateral lymph node metastasis, *n* (%)				0.0010
No	19 (95.0%)	13 (50.0%)	32 (69.6%)	
Yes	1 (5.0%)	13 (50.0%)	14 (30.4%)	
Central lymph node metastasis, *n* (%)				0.1512
No	16 (80.0%)	25 (96.2%)	41 (89.1%)	
Yes	4 (20.0%)	1 (3.8%)	5 (10.9%)	
Distant metastasis, *n* (%)				0.4291
M0	20 (100.0%)	24 (92.3%)	44 (95.6%)	
M1	0 (0.0%)	2 (7.7%)	2 (4.4%)	
Median preoperative serum calcitonin (CT) levels, pg/ml (Q1–Q3; range)	480.0 (87.0–1592.0; 12.4–11735.0)	400.0 (127.0–1200.0; 7.0–10968.0)	434.0 (92.5–1218.5; 7.0–11735.0)	0.7418
N-Miss	3	1	4	
Postoperative serum calcitonin (CT) levels, *n* (%)				0.0036
<2	14 (70.0%)	7 (26.9%)	21 (45.7%)	
≥2	6 (30.0%)	19 (73.1%)	25 (54.3%)	
(Stimulated) Postoperative serum calcitonin (CT) levels, *n* (%)				0.0036
N-Miss	0	7	7	
<2	14 (70.0%)	7 (26.9%)	21 (45.7%)	
≥2	6 (30.0%)	19 (73.1%)	25 (54.3%)	

Initial response to therapy, *n* (%)				0.0374
Excellent	14 (70.0%)	10 (38.5%)	24 (52.2%)	
Biochemical incomplete	6 (30.0%)	14 (53.8%)	20 (43.5%)	
Structural incomplete	0 (0.0%)	2 (7.7%)	2 (4.3%)	
Final follow-up, *n* (%)				0.0053
NED	14 (70.0%)	9 (34.6%)	23 (50.0%)	
Biochemical incomplete	3 (15.0%)	14 (53.8%)	17 (37.0%)	
Structural incomplete	1 (5.0%)	1 (3.8%)	2 (4.3%)	
Death MTC-unrelated	0 (0.0%)	2 (7.7%)	2 (4.3%)	
Death MTC-related	2 (10.0%)	0 (0.0%)	2 (4.3%)	
Additional therapies, *n* (%)				0.0293
No	18 (90.0%)	16 (61.5%)	34 (73.9%)	
Yes	2 (10.0%)	10 (38.5%)	12 (26.1%)	
Germline *RET* mutations				0.02321
No	15 (50.0%)	15 (50.0%)	30 (65.2%)	
Yes	5 (31.2%)	11 (68.8%)	16 (34.8%)	

IHC, immunohistochemistry; MTC, medullary thyroid cancer; TNM, tumor node metastasis; NED, no evidence of disease.
