# Supplementary material for: CD276 as a Candidate Target for Immunotherapy in Medullary Thyroid Cancer
Source: Int J Mol Sci. 2023 Jun 12;24(12):10019. doi: 10.3390/ijms241210019 (PMC10298428; doi:10.3390/ijms241210019)
Supplement: Supplementary file 1 [file ijms-24-10019-s001.zip › Supplementary Table S2.pdf]

**Supplementary Table S2. The hierarchization of variables related to final follow-up and initial response to therapy.**

| | | Initial response to therapy (less than excellent response) | | | Final follow-up (no remission of the disease) | | |
|---|---|---|---|---|---|---|---|
| | | n=22 | | | n=23 | | |
| | | univariable OR | 95% CI | *p*-value | univariable OR | 95% CI | *p*-value |
| Gender | Female | Ref. level | | | Ref. level | | |
| | Male | 2.08 | 0.59-7.29 | 0.2538 | 2.77 | 0.76-10.05 | 0.1214 |
| Age at diagnosis, years | | 0.98 | 0.95-1.03 | 0.4627 | 0.98 | 0.94-1.02 | 0.2462 |
| Tumor size, mm | | 1.03 | 0.98-1.07 | 0.2315 | 1.02 | 0.98-1.06 | 0.3183 |
| Multifocality | No | Ref. level | | | Ref. level | | |
| | Yes | 1.13 | 0.32-3.98 | 0.8452 | 2.31 | 0.63-8.47 | 0.2049 |
| Extrathyroidal extension | No | Ref. level | | | Ref. level | | |
| | Yes | 2.3 | 0.19-27.3 | 0.5094 | 2.1 | 0.18-24.87 | 0.5579 |
| Angioinvasion | No | Ref. level | | | Ref. level | | |
| | Yes | 1.74 | 0.26-11.51 | 0.5673 | 1.57 | 0.24-10.44 | 0.6378 |
| Central lymph node metastasis | No | Ref. level | | | Ref. level | | |
| | Yes | 0.7 | 0.11-4.64 | 0.7116 | 1.58 | 0.24-10.44 | 0.6378 |
| Lateral lymph node metastasis | No | Ref. level | | | Ref. level | | |
| | Yes | 7 | 1.61-30.45 | 0.0095 | 11.45 | 2.17-60.56 | 0.0041 |
| Preoperative serum calcitonin (CT) levels, pg/ml (groups) | up to 400 | Ref. level | | | Ref. level | | |
| | more than 400 | 1.32 | 0.4-4.38 | 0.65 | 0.76 | 0.23-2.51 | 0.65 |
| Germline RET mutations | No | Ref. level | | | Ref. level | | |
| | Yes | 2.5 | 0.72-8.71 | 0.1502 | 5.18 | 1.34-20.06 | 0.0172 |
