# Supplementary material for: CD276 as a Candidate Target for Immunotherapy in Medullary Thyroid Cancer
Source: Int J Mol Sci. 2023 Jun 12;24(12):10019. doi: 10.3390/ijms241210019 (PMC10298428; doi:10.3390/ijms241210019)
Supplement: Supplementary file 1 [file ijms-24-10019-s001.zip › Supplementary Table S3.pdf]

Supplementary Table S3. Comparing sensitivity, specificity, positive and negative predictive values and accuracy for CD276 immunohistochemistry and lateral lymph node metastasis in group with less than excellent response to initial therapy.

	CD276-positive group with less than excellent response to initial therapy	Group with lateral lymph node metastasis and less than excellent response to initial therapy	p-value
	n=22	*n*=22	
Sensitivity	72.7%	50.0%	0.0253
Specificity	58.3%	87.5%	0.0196
Positive predictive value	61.5%	78.6%	0.1309
Negative predictive value	70.0%	65.6%	0.5378
Accuracy	65.2%	69.6%	0.6565
