# Supplementary figures and images for: CD276 as a Candidate Target for Immunotherapy in Medullary Thyroid Cancer
Source: Int J Mol Sci. 2023 Jun 12;24(12):10019. doi: 10.3390/ijms241210019 (PMC10298428; doi:10.3390/ijms241210019)

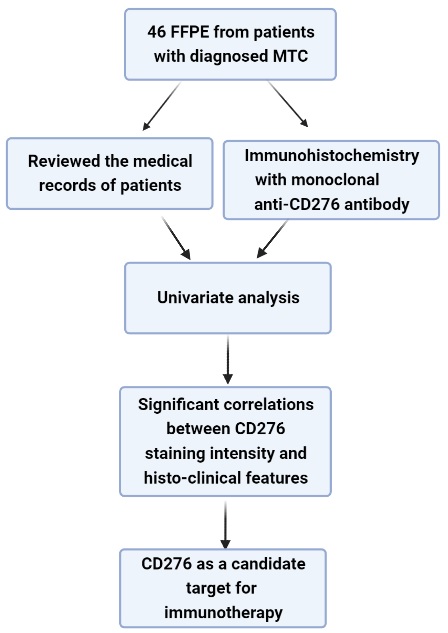

Supplement: Supplementary file 1 [file ijms-24-10019-s001.zip › Flow diagram.jpg]
